# Skill Breakdown and Symbolic Relating: A Functional Contextual Exploration of Choking in Sport

**DOI:** 10.3390/bs16071052

**Published:** 2026-06-24

**Authors:** Sara T. Svoboda, Patrick Smith, Denise M. Hill, Jamie B. Barker, Karl J. Steptoe

**Affiliations:** 1 School of Sport, Exercise and Health Sciences, Loughborough University, Loughborough LE11 3TU, UK; s.t.svoboda@lboro.ac.uk (S.T.S.); j.b.barker@lboro.ac.uk (J.B.B.); 2 Department of Psychology, University of Nevada, Reno, NV 89557, USA; patrick@coachpatricksmith.com; 3 Department of Sport and Exercise Sciences, Swansea University Bay Campus, Crymlyn Burrows, Skewen, Swansea SA1 8EN, UK; denise.hill@swansea.ac.uk

**Keywords:** choking in sport, functional contextualism, contextual behavioural science, relational frame theory, acceptance and commitment therapy, third-wave cognitive behavioural approaches

## Abstract

Choking in sport is both a highly researched phenomenon and a colloquial term for performance collapse, which has been at the centre of much debate. The past four decades of study continue to present challenges with its definition that have stifled progression with both research and applied intervention. This study adopted a functional contextual approach, with the aim of exploring how conceptualising choking through this lens might better serve practitioners and researchers, to build more impactful and meaningful processes for behaviour change. A purposive sample of 12 athletes, who identified as having experienced choking, took part in one of four focus groups. Thematic analysis provided four themes that explain choking from this functional contextual perspective, as a process of creating maladaptive personal narratives, responding to symbolic cues, rule-following, and strategies to alleviate the discomfort of challenging inner experiences associated with the experience. Findings provide preliminary support for functional contextualism as a worthwhile research lens for choking, with the suggestion that returning to observable definitions of choking may offer practitioners greater insight into relational processes underpinning choking.

## 1. Introduction

The experience and study of choking in sport continues to plague athletes and researchers alike. However, over four decades of exploration into this performance phenomenon have failed to yield a universally accepted definition and inform meaningful and sustainable interventions that promote change. Current definitions are problematic and have received criticism for conflating causes with the phenomenon, leading to circular reasoning (Jackson, 2013). For example, Mesagno and Mullane-Grant (2010) defined choking as being “caused by an elevation in anxiety levels under perceived pressure” (p. 343). In response to criticism, Mesagno and Hill (2013) issued a call to action within the field, contesting that “the foundation of choking research may have been developed from misguided definitions, which need to be revisited” (p. 7), a view supported by researchers in the field (Jackson, 2013; Buszard et al., 2013). This lack of consensus is considered to have stifled progression and led to a paucity of pragmatic and long-term solutions to alleviate the choke (Christensen et al., 2015; Gray, 2020; Hill & Shaw, 2013). Third-wave cognitive behavioural research suggests that solutions for choking under pressure that equip performers with the skills to change how they relate to what they think and feel, rather than changing what they think and feel, may offer more sustainable strategies for optimising performance under pressure (Mesagno et al., 2020).

Conceptualisations of choking are predominantly reflective of the mechanistic approach that has dominated the research and have focused on identifying mechanisms and moderators believed to underpin the experience. These include the presence of debilitating anxiety (Beilock & Gray, 2007; Hardy et al., 1996), a maladaptive focus of attention (Baumeister, 1984; Carver & Scheier, 1981), increased reinvestment (R. S. Masters et al., 1993; R. Masters & Maxwell, 2008), or the perception of insufficient resources to meet the demands of the situation (e.g., skill, support; Hill et al., 2009). These conceptualisations have largely been arranged around two explanatory models: distraction and self-focus, which contest that choking is the result of disrupted attention due to heightened anxiety, and have been used to explain the mechanisms by which novices and experts choke, respectively. Interventions to address distraction explanations for choking include pre-performance routines and distraction training, while quiet eye training (Vine et al., 2013), neurofeedback (Ring et al., 2015), task-irrelevant cues (Balk et al., 2013), and anxiety acclimatisation training are examples of self-focus applications (see Gröpel & Mesagno, 2019 for full review). Traditional choking interventions have been criticised for testing only short-term effects and using samples of trained novices, club, or collegiate participants rather than elite samples, which has led to a limited understanding of long-term intervention implications and how they may aid performers at the highest levels (Gröpel & Mesagno, 2019).

A review by Mesagno et al. (2020) of applied sport psychology interventions showed that the majority have been representative of a traditional cognitive behavioural approach. The authors suggested that traditional strategies may be counterproductive in achieving goals of enhanced performance, as efforts to remove or reduce unwanted psychological experiences (for example, heightened feelings of anxiety or negative thinking) may be exacerbating their effects on performance (Mesagno et al., 2020). ‘Ironic rebound effects’ (also referred to as ‘ironic mental processes’; Wegner, 1994, 1997) is one theory that may explain the pitfall of traditional interventions that focus on altering the content of unwanted inner experiences, for example, Psychological Skills Training (PST) techniques such as cognitive restructuring (Mesagno et al., 2020). While the majority of choking explanations and interventions have prescribed to traditional cognitive-behavioural approaches, research efforts from the third-wave cognitive-behavioural tradition suggest that contemporary interventions such as mindfulness and acceptance strategies are better able to influence the performance experience through mechanisms that are fundamentally different to traditional methods (Gardner & Moore, 2017; Birrer et al., 2012). Such approaches function to enhance mental efficiency by promoting full awareness; in-the-moment acceptance of one’s internal states; task-focused attentional processes; and “values-driven” commitment to behaviour (Gardner & Moore, 2017, p. 2). The present study targeted a methodological departure from traditional second-wave approaches for studying choking in sport, as to date, these have not provided conceptual consensus or offered efficacious applied strategies.

### 1.1. A Functional Way Forward for Choking Research

To address shortcomings of traditional cognitive-behavioural approaches in both the operationalisation of, and intervention for the choking experience, this study adopted a new philosophy. Contextual Behavioural Science (CBS) is a scientific paradigm grounded in behaviour analysis and a pragmatic worldview known as Functional Contextualism (FC). This is the notion that the function of any behaviour (the reasoning behind its occurrence) is dependent on, and changes with the context it is situated within (both historical and situational; Twohig & Levin, 2017). Traditional cognitive approaches to choking research, which belong to the mechanistic camp, dictate that a model for explaining behaviour is ‘true’ insofar as there is correspondence between the model and the world as it actually is. Based on this criterion, a choking theory is only considered ‘true’ to the degree its predictions match the data (Hayes et al., 1988). Conversely, within contextual behavioural analysis, analyses are evaluated through the pragmatic truth criterion. This criterion stipulates that a conceptualisation is ‘true’ when enabling both predicting and guidance as to how to influence behaviour (Twohig & Levin, 2017). CBS focuses on the role context plays in understanding and influencing human behaviour to develop basic behavioural accounts of complex actions, and to apply these advances to alleviate human suffering (Hayes et al., 2003) (for a review of CBS, see Zettle et al., 2016).

### 1.2. RFT: A Framework for Achieving a Functional Contextual Understanding

Relational Frame Theory (RFT) potentially offers a more helpful understanding of contexts underpinning choking as a complex human behaviour. RFT provides a functional–analytic perspective which proposes that psychological issues are largely underpinned by tendencies and faults within human language and cognition. The theory postulates that the core of these processes is the learned ability to form mutual relationships among events, combine these into relational networks, and to change the function of related events (Hayes, 2016). RFT explains how we learn and make sense of our world by relating to our internal and external experiences. Within RFT, ‘verbal behaviour’, also referred to as ‘arbitrary applicable derived relational responding’ (AADRR), is discussed as the human ability to symbolise. Based on contextual cues, humans can derive relations between virtually anything, including events, experiences, and people (Bennett & Oliver, 2019). This relating occurs in a variety of ways through relational framing—a process of relating stimuli to one another based on context, rather than physical properties.

RFT explains how the human ability to relate through processes of social learning, language and rules plays a crucial role in impacting our subsequent behaviour (Twohig & Levin, 2017). Humans did not evolve automatically programmed to understand ‘anxiety’ as something inherently negative; it only acquires meaning through the arbitrary social context that is created around it. For example, experiencing anxiety (perhaps somatically through a racing heart and increased adrenaline) is quite adaptive in a context when faced with a threatening wild animal. In this instance, it could be framed as helpful, adaptive or ‘good’ for initiating an escape, as increased oxygen is delivered to working muscles and the fight or flight response is triggered. It is only through socialisation and what anxiety becomes related to within complex relational networks that it becomes something people identify as aversive or seek to avoid. RFT explicates a process whereby the way people relate to their experiences alters the function of these experiences. From a choking perspective, this can be conceptualised as the way athletes think about their inner and external experiences, and is known as the transformation of stimulus functions (Twohig & Levin, 2017). The human ability to relate is a double-edged sword: it both facilitates learning but also becomes consequential when it is applied in an unhelpful context where it does not serve purposes necessary for performance.

### 1.3. Third-Wave Approaches for Facilitating Performance Under Pressure

The emphasis on changing the *function* opposed to *form* of behaviour is a foundation of Acceptance and Commitment Therapy (ACT), a third-wave cognitive-behavioural therapeutic/training model, which operates as an experiential therapy wherein processes of psychological flexibility are utilised to alter athletes’ responses to their internal experiences, instead of changing or reducing the experiences. In this way, the goal of work that typifies interventions belonging to the cognitive-behavioural tradition for alleviating choking under pressure, is shifted from “targeting specific cognitions and emotions to alter their downstream effects on other behaviours,” for example, cognitive restructuring of negative thoughts to induce optimal mood states, to altering the *context* in which these behaviours occur; for example, experiencing anxious thoughts in anticipation of an important competition in the context of acceptance of the ebb and flow of internal experiences in sport (Twohig & Levin, 2017, p. 752). ACT posits six processes that allow athletes to cultivate a posture of openness, engagement, and awareness as they *perform with* challenging or unwanted psychological experiences. The six processes of ACT that work in synergy to encompass ‘psychological flexibility’ include: acceptance of emotions; defusion from thought processes; present moment awareness; flexible perspective-taking (or self-as-context); committed action; and identification of values.

Discrepancies across the literature regarding the nature of choking in sport, including how the experience is conceptualised through qualitative studies (Hill et al., 2010b, 2017, 2019; Hill & Shaw, 2013; Hill & Hemmings, 2015; Hodge & Smith, 2014), has also limited progress in developing intervention solutions. The purpose of the current study is to gain understanding of the choking phenomenon through a functional contextual lens. The specific intention is set to enable development of a more pragmatic conceptualisation of choking in sport, and provide a foundation for future applied research and the development of more effective interventions that positively impact this unique performance experience. Exploring an alternative conceptualisation is intended to extend beyond description to better inform intervention and meaningful behaviour change which offers the opportunity to better predict and influence the choking experience. Applying CBS as a new research lens to a well-developed research area offers the advantage of deriving greater contextual understanding of this phenomenon. We will explore the experience of choking through the accounts of athletes to answer the research question: how might the experience of choking in sport be conceptualised through a functional contextual lens?

## 2. Materials and Methods

### 2.1. Research Design and Methodology

The study is informed by a functional contextualist philosophy that emphasises two elements of behaviourism: (1) behaviour can only be understood in relation to the context it occurs in; and (2) for behaviour to be influenced or understood, its function must be studied (Törneke, 2010). A pragmatic epistemology is adopted where the value of knowledge lies in its practical application and utility in predicting and influencing behaviour. From an ontological perspective, functional contextualism adopts a behaviour-analytic perspective where meaning is derived from the ongoing interaction between behaviour and the environment.

Relational Frame Theory (RFT; Barnes-Holmes et al., 2002) was adopted as the theoretical framework to explore the choking phenomenon. This lens informed the contextual and behavioural focus of the focus group interview discussion in addition to deductive analyses. The research question was explored qualitatively through focus groups, which allowed for insight into the experiences of athletes who have choked under pressure, with a focus on their accounts of physical behaviour in context and verbal behaviour, for example, their unique history of relational framing.

### 2.2. Participants

Purposive sampling was utilised to ensure focus groups were populated with athletes who self-identified as having experienced choking in sport. Athletes who experienced a choke (acutely, chronically or episodically) and who competed at a competitive level (national, international, professional, university performance sport, club, or regional) were eligible for the study. Inclusion criteria stipulating athletes competed at a high level was imperative for athletes to draw from high-stakes/competitive performance contexts and to ensure that they had an understanding of what level of performance they were typically capable of, when choking did not occur. A total of four focus groups were conducted by the first author, ranging between two to four participants per group. Researchers attempted to remain consistent with Krueger and Casey’s (2009) focus group guidelines which recommend sampling approximately three to five focus groups, consisting of approximately four to eight participants per group. Due to logistical and recruitment challenges, however, the final sample included one focus group of four participants, two focus groups consisting of three participants and one with two participants. Based on previous qualitative choking literature that included a sample range of five to 12 participants (Gucciardi et al., 2010; Hill et al., 2010a, 2017), this was deemed a sufficient sample for the present study. The participant pool included athletes (*n* = 12) aged between 18 and 62 years (*M* = 25.42 years, *SD* = 11.84). The sample consisted of seven female and five male participants. The female participants included international-level athletes (*n* = 3), national-level athletes (*n* = 3), and one athlete competing at the university/regional level. The male sample consisted of one athlete competing at the international level, two males who competed at the semi-professional level, and the remaining two male athletes competing at the regional county or club level. The sports represented in the sample were international-level athletics and weightlifting; national-level long-distance running and swimming; professional-level football and cricket; and county-/regional-level golf, gymnastics and rugby union.

### 2.3. Procedure

Ethical approval for the study was granted by the Loughborough University Ethics committee (LEON; project ID: 6405). To remain consistent with the assertion that research has been stifled by highly contested definitions of choking that tend to reflect a mechanistic bias, a detailed definition of what constitutes a choke was purposely omitted from the recruitment documents. Instead, participants were recruited if they identified that they had experienced choking based on a broader behavioural understanding of the experience, for example, experiencing debilitating skill breakdown in a high-pressure scenario/environment and having a history of being capable of delivering performance under such conditions previously. The researchers managed the potential subjectivity introduced into the data through participants’ self-identification with the choking phenomenon, through iterative analysis of participants’ account of their choking experience. We have argued that current definitions of choking are not fit for purpose; therefore it was a strategic and deliberate decision to allow participants’ accounts to be at the centre of analysis. Participants who contacted the first author were emailed the study information and informed consent document which they were asked to read, sign and return prior to participation.

Participants were reminded of their right to withdraw and a process that would maintain confidentiality throughout the study. The purpose of the study was reiterated, specifically the aim of learning about athletes’ experiences of choking under pressure, with special attention paid to how this experience might be conceptualised regarding the contexts the experience was situated in. Despite the small sample size, based on qualitative methods in the literature (Braun & Clarke, 2013; Charmaz, 2006; Creswell & Poth, 2016), four focus groups was considered sufficient and saturation was deemed to have occurred when preliminary iterative analysis did not produce any new codes or reveal new theoretical insights. Authors engaged with an iterative analysis process that involved taking notes and memos of initial interpretations and emerging insights to inform their sample decision.

The focus groups were audio- and video-recorded to aid transcription as well as capture in situ focus group participant behaviour, which is consistent with the CBS perspective that both external and verbal behaviour are central to understanding behaviour. Three of the four focus groups took place virtually and were conducted via the video conference calling software Zoom (version 5.0). The remaining focus group took place in person.

### 2.4. Semi-Structured Focus Group Interview

The interview schedule structure was partly informed by Hill et al. (2010a) and was piloted with a four-athlete focus group to ensure athletes were probed comprehensively on their experience of choking. This entailed incorporating factors examining the perceived antecedents of the choke, the experience of the choke with respect to cognitions, emotions and physiological sensations, and consequences of the choke. The first section aimed to generate general discussion around a choke, including how they identified the experience distinct from other performance collapses and any beliefs regarding what constituted a choke. The second section allowed participants to reflect on their personal experience of performing under pressure, including what types of pressurised environment they had previously faced. The third section asked participants about antecedents relating to performing under pressure. Section four probed participants about the experience of the choke (thoughts, emotions, and physiological sensations), while section five asked participants to reflect on the consequences with a particular focus on the internal (thoughts and emotions) and external (overt behaviours following the choke) to further draw out understanding of the contexts surrounding the experience. Section six prompted participants to reflect on strategies they had previously employed to recover, and the final two sections asked participants to summarise their experience and contribute any additional information. To explore the context surrounding the experience, participants were asked to reflect on the internal (thoughts, emotions and sensations), situational (their history of performing in similar situations) and environmental (both internal and external) contexts of their experiences.

### 2.5. Data Analysis

Focus group data were inputted into the transcription tool Otter (otter.ai). To ensure accuracy, the first author checked the transcription twice against the audio and video recording. The transcripts were uploaded into the coding software NVIVO version 12 to assist with thematic analysis. The first author conducted a thematic analysis consistent with the six phases outlined by Braun and Clarke (2006). The flexibility of thematic analysis enabled the identification of reoccurring themes with respect to how choking behaviours function in different sport contexts as well as patterns in verbal behaviour.

The researcher adopted a philosophical orientation informed by CBS, where participants’ accounts of their behaviour were analysed with a focus on the function of behaviour in context. The theoretical assumptions of RFT informed a predominately deductive analysis as researchers were interested in acquiring an understanding of the choking experience through a CBS lens. Phase one entailed familiarising with the transcript by checking it against the video and re-reading several times. Phase two involved coding data that the first author identified as relating to the core components of RFT (for example, contextual cues, relational frames, and rule-governed behaviour) or the applied psychological processes of ACT (for example, instances of cognitive fusion, self-as-content or experiential avoidance) which would contribute to the conceptualisation of choking through this specific lens.

Data were coded at the semantic and latent levels, where both the participant’s explicit description and conceptualisation of choking events and performance under pressure were preserved in the code names, whereas latent codes were utilised to communicate the aspects of the experiences as interpreted by the first author that reflected aspects of RFT or ACT. A quality of CBS research that demarcates itself from other scientific paradigms is that, when analysing behaviour, the behaviour of the ‘whole’ organism must be considered. To conduct analysis consistent with the CBS approach, the first author retained non-verbal language and behaviour within coded extracts to provide theoretically-consistent insights into the experiences of choking under pressure. Retaining non-verbal behaviour including gestures, body language, and properties of speech allowed the researcher to acquire a comprehensive understanding of the choking experience as recounted by the participants. The focus group interview represents one such context to observe avoidance behaviours, cognitive fusion and a lack of present moment awareness through participants’ retelling of their choking experiences (Hayes et al., 2004). This method of highlighting observable behaviour allows for ‘thicker descriptions’ and interpretations regarding the experience of choking under pressure, when compared to the collection of only verbal language (Denham & Onwuegbuzie, 2013). This was captured in quotations via double brackets, for example, ((hands raised to cover face)) or ((moved body away from screen)). Following the coding phase, the first author employed phases three (searching for themes) and four (reviewing themes).

### 2.6. Methodological Rigour

The rigour of data analysis was strengthened through researcher triangulation. The second, third and fifth authors served as ‘critical friends’ during theme generation to challenge the findings and construction of knowledge (B. Smith & McGannon, 2018). The third author offered feedback on the interpretations of the participants’ conceptualisations of choking during theme development. The second and fifth author provided feedback with respect to deductive analysis of the data as it related to RFT as the underpinning theoretical framework, for example, when organising codes into sub-themes, which reflected an RFT-perspective applied to the choking experience. With respect to bias, researchers must acknowledge how perspectives brought into qualitative investigation may affect how the data are analysed, interpreted and reported (Sparkes & Smith, 2013). At the time of data collection and analysis, the first author was a 27-year-old elite athlete who had played competitive sport in North America/represented a North American country, in addition to competing professionally within the United Kingdom. The author also identified having experience performing under pressure and had personal experience of choking. Additionally, the first author was undertaking training as a sport and exercise psychologist, which includes practicing through an ACT-informed approach. Because of this position, the author acknowledges that the analysis is subject to bias and subjectivity with respect to the questions posed, interpretation of the responses and the construction of knowledge. To manage this, the author engaged with reflexive journaling throughout the study to make habit of scrutinising their position within the data, through continuous questioning of ‘what I know’ and ‘how I know it’ (Hertz, 1997; S. Smith, 2006). Additionally, the involvement of the third and fourth authors who practice sport psychology through a traditional cognitive-behavioural approach (for example, CBT and REBT) were included as critical friends. While this inclusion of critical friends does not eliminate biases, it does provide the reader transparency and awareness with respect to the author’s impact on how data were analysed and how conclusions take shape.

## 3. Results

The purpose of the study was to explore the experience of choking under pressure with the aim of moving towards a functional contextual understanding that goes beyond description and better informs intervention and meaningful behaviour change. This was addressed through the research question: how might the experience of choking be conceptualised through a functional contextual lens? A combination of deductive and inductive thematic analysis was used to identify four main themes that conceptualise the experience of choking: personal narratives of choking; conditions for the choke; rules for choking; and unworkable strategies to alleviate the choke. The four master themes consisted of 27 higher-order sub-themes, which were generated from 61 lower-order themes, which in turn were abstracted from 179 raw data codes. To demonstrate this process, the extract: “there’s, all sorts of stuff whizzing around my head ((circles finger around head, resting hand on head)). That shouldn’t be there ((shaking head))” was coded as ‘self-rules for how you should think, feel and perform’ and was subsequently grouped with other codes to form the ‘fixation on rules for performance success’ lower-order-theme. This was then encompassed by the ‘consequences of rule-following’ higher order theme, and eventually, the ‘rules for choking’ master theme.

Evidence for the themes is provided by quotations extracted from the semi-structured focus group interviews. Initials are used to denote the competition level (C = competitive; I = international; N = national; and P = professional) and gender (M/F) of the speaker. Pseudonyms were also assigned to participants and are used within extracts.

### 3.1. Personal Narratives of Choking

Personal narratives of choking was identified as a main theme that encompassed the way athletes perceived and conceptualised their experiences through reflections of choking under pressure. This theme comprised ten sub-themes developed from 69 raw data codes (see Table 1). Through a contextual behavioural lens and RFT framework, the sub-themes illuminate the various verbal processes athletes engaged in during their experience of performing under pressure. The sub-theme ‘attachment to self-stories,’ captured athletes’ experiences of establishing self-concepts of a ‘choker’ reflected by absorbing experiences into a sense of self and which was an internal experience widely acknowledged across participants: “It’s well-known in the swimming community that I train well. She can’t race. She just chokes. And it makes you feel a bit like, ‘oh, that’s what- it’s known. You’re known for that.” (Jamie, NF2). An international weightlifter echoed this narrative, reflecting that during her experience: “I would have these intrusive thoughts of, ‘it’s gonna happen again, this is just what you do ((laughs)) you just bomb out,’ even though I only did it once.” (Hannah, IF4). This quotation illustrated the tendency for athletes to absorb (even one-off) experiences into the sense of self, which then posed challenges for the remainder of the current performance and future performances. In his experience of choking, a footballer reflected on engaging in behaviours in response to a rigid identification with a ‘perfectionist’ self-label:

I’ve come into the game with a doubtful mind and a small niggle...when everything is falling...suddenly that problem [niggle] which I’d give a two out of ten in my mind becomes a *six* out of ten and I’m like, ‘okay, I just want to take myself out of the firing line, like sub me off, get me off the pitch...it’s just not working’...you doubt yourself, there’s a lot of attack on the self…I tend to beat myself up...I’m a perfectionist.

(Tony, PM1)

In this example, the footballer’s self-label of ‘perfectionist’ may have potentially precluded him from responding to intrusive thoughts of his injury, or the challenge of the match in an adaptive way (for example, requesting to have his injury assessed, or directing his attention to his next involvement to positively impact the match). Conversely, the athlete reflected that he responded by asking to be substituted out of the match (attempting to regain a sense of control over the situation to avoid a performance which was inconsistent with his sense of self).

Athletes’ narratives were also defined by a tendency for preoccupation with the future. This was demonstrated by a golfer who reflected that, “I know when I’m standing over the ball, it’s all gonna go wrong…before I’ve even taken the club backwards. So, I’ve already lost before I’ve started and that’s quite...horrible” (Paul, CM1). Narratives of future-oriented cognitions were also represented through grappling with negative imagery of performance, as articulated by a national-level cricketer:

Sometimes before an event, I’ll kind of be *seeing* myself bowl badly...and then I’ll try to counteract and try to *force* an image in my head...of seeing myself bowl *well*, so it’s that conflict in my head...do I try and focus on bowling? Seeing myself bowl well? Or do I sit with the feeling...the imagery of me bowling badly...thinking about what’s going to happen...instead of just being present and grounded...I’m in a different place.

(Izzy, IF2)

Athletes’ fixation on the future also extended to fears of failure; for example, an international weightlifter reflected on frequently experiencing the thought, “what if you bomb again?” (Hannah, IF4). This sentiment was supported by an international discus thrower who reported fixating on similar future-oriented cognitions laden with fears of failure: “...there was so much stuff going through my head like, what if *this* happens? What if I didn’t get through all three throws, or got three no-throws?... Didn’t get through to the final…?” (Tanisha, IF1)

The sub-theme ‘preoccupation with the future’ also captured the experience of athletes wherein the choke was conceptualised as an overwhelming fear held about not-yet-experienced, or never-before experienced events. For example, a gymnast shared a narrative of how competing in the vault (which had historically been her favourite event), functioned to evoke fear and consequently avoidant behaviour in the context of high pressure and a fear of failure: “I would then think of, ‘what if I really hurt myself here? What if I let my coach down? What if I’ll never be able to do it again?’” (Emma, IF3).

Athletes’ personal narratives of the choking experience made reference to problematic patterns of attention, which encapsulated the ‘consequences of inflexible attention’ sub-theme. Athletes often cited an experience of feeling overcome with a sensation of being ‘in their head’ or ‘out of body.’ This sub-theme reflected athletes’ experiences of disconnecting from the present moment (where performance is optimised as athletes can make moment-to-moment adjustments and decisions based on the changes in environment, opponents and demands). Athletes recounted experiences of grappling with thoughts of self-doubt, including an influx of uncertainties about trusting well-rehearsed processes that impacted their ability to remain engaged in the present:

Normally, I will put my hands on the bar, and be like, ‘one hand, two hand.’ As in I’m really present in what I’m doing, just so that it’s the same every time I do it. But then [reflecting on past choking experiences] I’m like, ‘should you move your feet? Should I do something...’ and I start thinking about this stuff. And I’m like, ‘what are you doing? Stop thinking about that.’ I think internal dialogue is a key indicator for me, like, ‘oh, no, this isn’t going too well.’

(Hannah, IF4)

The notion of this metacognition dominating over awareness was also articulated in the sub-theme ‘fixating on inner experiences,’ where athletes cited a tendency to engage in a struggle with their thoughts to be part of their choking stories. Athletes reflected that challenging thoughts, memories, and mental images, when excessively attended to, often coincided with their experience of a choke. For example, a semi-professional footballer reflected on his experience of choking, which he illustrated through the narrative of the ‘red mist.’ This metaphor referenced challenging thoughts, which in a context of judgement and fixating on internal experiences, functioned to support maladaptive behaviours under pressure:

It’s like an uncontrollable pattern of thoughts, where you struggle to just latch on to something and say, ‘that’s me, this is what I’m good at.’ You almost start doubting yourself in the middle of the pitch if you can perform basic functions…’ and that for me is like the red mist ((covers face with hands)) because you then start getting even more anxious. So even when you’re trying and you actually might be reacting positively to it, your mind is telling you that you should be doing way more...it just snowballs into a bad, really bad performance…you’re overthinking it, there’s a seed of doubt, which is now expanding, and, like almost warping your performance.

(Tony, PM1)

In this quotation and through a CBS lens, it is important to note the physical behaviour of Tony covering his face with his hands that accompanied his verbal recounting of his experience of the ‘red mist,’ which was articulated as a flurry of overwhelming self-doubts and criticisms of his performance. Athletes referred to experiences where, after having time to reflect, they suggested there was potential to engage in more flexible behaviours or responses to pressure as opposed to being at the mercy of their experiences. The sub-themes ‘belief that a choke is the result of failure to adaptively respond to warning-signs,’ ‘choking is about how you relate to what you experience,’ and ‘flexible responses to pressure are possible,’ represented conceptualisations of the experience where beliefs were held about athletes’ ability to recognise opportunities to engage with adaptive responses to pressure. For example, one athlete reflected:

There’s still a lot of emotions around the competition arena which can lead to a choke or not, in some cases...You still get the emotions pent up, but, it’s whether you can deal with them enough...I still think it’s very hit or miss whether I do or don’t after...haven’t quite figured out what makes it choke and what doesn’t because the emotions are normally still all there. Like around a choke.

(Claire, NF2)

These sub-themes expanded the choking narrative to include perspectives that performers are capable of adjusting their response to stimuli in their environment to achieve positive performance outcomes. Similarly, a professional cricketer likened the emotional and mental experience of the choke to optimal performance states (e.g., flow, clutch), contending that the context of internal experience (e.g., a blank mind in the context of self-criticism vs. non-judgmental awareness or self-compassion) is what differentiates performance outcomes:

For me, personally, it’s [choking] such a similar feeling ((laugh)). So *different*, but such a *similar* feeling of like performing at your best...when you’re performing at your best things just naturally happen, you’re not thinking things like that. And then when you choke, you’re *also* not thinking...you *can’t* think if you wanted to, you start *trying* to think...it all escalates... I think that’s one of the things that’s hard to get my head around. The fact is, you know, *so similar*, but so impossibly *different*.

(Rob, PM2)

The juxtaposition reflects how the point of difference between excelling and collapsing through experiences of a quiet mind relates to the context around how it is related to or symbolically ‘framed’ by the athlete. In this example, the athlete articulates how ‘not thinking’ functions to facilitate flow and a sense of calm in a context of being aware of and open to one’s internal states, but functions to elicit panic in a context of a need to control and change one’s cognitions when performing under pressure.

Within the ‘flexible responses to pressure are possible’ theme, the notion of athletes having the capacity to exercise control over their performance experience was reflected by narratives of athletes who reported engaging in mindful- and acceptance-based strategies (for example, mindfully committing to their pre-competition routine or adopting an attitude of acceptance towards the flux of internal experiences). An international weightlifter shared her experience of engaging in flexible responding under pressure: “Recognising that I’m having these thoughts but trying to breathe out, having this feeling of closure. I’m letting go of those thoughts. That’s something that I will try to do a lot in-the-moment.” (Hannah, IF4)

### 3.2. Conditions for the Choke

Conditions for the choke consisted of nine subthemes (see Table 1). The ‘context of ‘this matters’ created around the event’ sub-theme was reported across athletes where conditions of heightened importance of an event preceding choking were recognised as an antecedent for the choke.

If you see me down the driving range, I look like a half decent club golfer. I hit it quite well, 99 times out of 100. And it’s effortless. Swing is effortless. I don’t think about it. It’s instinct. But when ((pauses and laughs)) but *when it matters*, when the golf ball was on the golf course ((widens eyes)) there’s something weird going on in my head, that doesn’t allow me to replicate what I know is the normal, normal way of hitting a ball, it’s really weird.

(Paul, CM1)

The salience of a competition’s importance was also reflected in the experience of creating a context of ‘this matters’ which interfered with their mindset approaching competition: “Playing against the team one place above us...whoever won would get promoted...I think being aware of this...I could have told you that I was not going to be able to perform the night before...because of the pressure of the game” (Rob, PM2). The same athlete indicated that this context was also created through verbal cues in the performance environment: “Everyone’s body language and things change [before high pressure matches]…for us it’s from the people that come watch us…the committee at the club. They go from not talking to you ((smiles)) to saying, ‘big game this week!’”

Conditions for the choke were also represented by the sub-themes ‘low self-trust’ and ‘threat mindset’ which were believed to provoke the choke. Athletes articulated a tendency to perceive competition as exceeding their ability to cope (e.g., “too big for me”)—often with respect to demands, necessary resources, and competition stakes. For example, one athlete acknowledged thoughts he experienced after choking in a cricket match: “It doesn’t really matter what someone sees. For me, I know, internally, *you know* you’ve choked, and you’ve known that the game is *too big* for you. And your performance” (Rob, PM2). Similarly, a golfer who reported chronic episodes of choking acknowledged a similar sentiment, reflecting that: “When I’m playing golf, it’s *too big* for me now. Can’t do it anymore” (Paul, CM1). Participants reported common consequences of relating to their performance in this way as abandoning workable behaviours or strategies in service of extinguishing their anxieties of inadequacy in meeting competitive demands:

A couple of years back after I’d put this routine in place...I’d hit a certain amount of balls before a game, did a certain amount of visualisation. [On the day of the choke] I spent an extra ten minutes out in the middle doing visualisation in the morning…and I hit probably 20–30 extra balls in the net, because I was like, ‘ah, it’s a massive game. *More, more, more.* Get yourself ready. Come on.’ And I’m out of—I’m out of that routine that has served me so well and that I’ve tried to develop so that *I don’t do this*...and actually, it’s making it worse because now I’ve got a routine.

(Rob, PM2)

The sub-theme ‘historical influences for the choke’ articulated the way past experiences performing under pressure impacted participants’ current mindset when approaching competition. Participants identified a tendency to relate present experiences to past choking experiences as historical contexts for the choke. Several athletes reflected on emotions that resurfaced in competitions which bared similarity to past events, as well as physiological sensations, somatic and cognitive anxiety, panic, and low confidence, which they associated with an impending choke; for example:

When I think about my experiences, I always think about that tightness in my throat...that was one of the tell-tale signs…tightness in my throat, tension in my shoulders and then it was so hard to get that confidence back…as soon as I felt like that…I felt like uh it’s going to happen again kind of thing.

(Izzy, IF2)

This notion of relating physical or emotional states and the choke was also evident in the sub-theme ‘relating to anxiety as bad predicts maladaptive behaviour,’ and was identified as examples of both causal and coordination relational framing:

As soon as I wake up on the day of the race...I get physical symptoms of, ‘I *know* it’s gonna happen.’...At the start of the race, I can kind of know like in the warmup, how I’m feeling and be like, ‘Oh, I’m feeling quite tired.’ If I know I’m feeling tired in the water when I know my body is technically rested ((air quotes)). I know that I’m probably going to choke.

(Jamie, NF2)

### 3.3. Rules for Choking

This theme encapsulates three lower-order themes (see Table 1). The sub-theme ‘self-rules that anxiety and responses to pressure need to be controlled’ explores an implicit form of rule-following where participants made references to responding to their own experiences of anxiety around performance with judgement and restrictions around what they ‘ought’ to be feeling (e.g., ‘too much anxiety,’ feeling a sense of panic around one’s experience of pre-competition anxiety or dwelling on one’s emotional state before competition). These rules put participants in contact with the consequences for performance as they prioritised fixating on and adjusting emotional responses to pressure, rather than focusing on cues or relevant aspects of their performance environment that could help facilitate optimal performance. For example: “It would be in the sense of panic or confusion as to why I feel that? Why don’t I feel more up for it, or why aren’t I feeling ready, or why don’t I feel how I normally feel?” (Calin, IM1). Similarly, a golfer reflected on his internal states: “I feel really uncomfortable over the ball. There’s, all sorts of stuff whizzing around my head ((circles finger around head, resting hand on head)). That *shouldn’t be there* ((shaking head))” (Paul, CM1).

This sub-theme, which captured a widely acknowledged experience of fixation on rules for performance success, included rules and narrow parameters surrounding how one should think, feel, and perform for optimal outcomes. Several rules were implicitly identified in participants’ responses, and included a need to ‘prove oneself,’ at a higher level of competition or needing to make a competition ‘worth it,’ based on extensive training and preparation devoted to the sport. Athletes reflected on implicit rules for performance, including a need to strike a balance between excitement and anxiety. In the second sub-theme, ‘negative effects of language in the performance environment,’ participants described the effects for performance under pressure, as it was identified that athletes tended to fixate on the content of language prior to the choke. For example, one swimmer reported that in the situational context of a high-pressure environment and an internal context of low self-trust, her coach’s pre-race instructions provoked dysfunctional behaviour. In these contexts, the athlete relinquished trust in her instinctual knowledge for how to execute her race plan, and instead, fused with pre-race instruction (which typically served a function of highlighting tactical cognitions to influence behaviour in training), which instead was perceived as a rule to be followed:

What is said in that pre-race chat…I will hyper-fixate on what he said [coach]. So, if they say, ‘easy speed,’ I’ll fixate on the wrong part and go with the speed part, then it’s not easy. And then if they say, ‘go out relaxed,’ sometimes it’s a bit *too relaxed*…when I know I do it in training all the time, but not thinking about it, like I *know* how I’m meant to swim it…my body *knows*. I don’t need the whole, ‘focus on this, this, this,’ because I already know it. And if I hear that, I’m going to hyper-fixate on it too much and that’s gonna stress me out.

(Jamie, NF2)

Expectations for performance were also cited as forms of implicit rule-following behaviour underpinning the choking experience. Several participants identified the experience of low perceived pressure, coupled with high expectations to perform, either due to low challenge presented by the opponent or optimal environmental conditions to succeed (e.g., ball lying nicely on a green), which paradoxically created implicit pressures for performance success:

There was no real- no real pressure going into it as such. So yeah, it was it was kind of expected...There were competitions where you’re...expect- it’s yours to lose, like people are expecting...But this one was like, as long as I just turned up and made 90% of my lifts I would win. So, it wasn’t even like *mine to lose*, like something seriously bad had to happen in order for me to *not* win.

(Calin, IM1)

The quotation reflects the commonly held view that choking often occurs in the context of an expectation to perform. Participants reflected on those environmental conditions that supported favourable performance outcomes (e.g., easier competitive field, optimal playing conditions, favourable scenario in the game/event) and an expectation that athletes would be able to meet such demands, often functioned as rules for performance (e.g., you ought to, you should, you must/there’s no reason why you shouldn’t do well here). Conversely, some participants reflected that they felt protected against the choke when expectations to perform were relatively low (e.g., due to poor conditions, more challenging opponent) and that their performance was freed in a way that often facilitated performance:

If you’ve got a normal stance, you’re on the tee... there’s nothing in your environment that should prevent you from hitting a decent shot. So, your expectations are high. But when you’ve got a hideous stance or you’re in the rough or something, you know, my golf course we have a lot of heather and it’s really hard to make decent contacts with the ball...but invariably, I *do* in those situations because...maybe that is it, maybe this is about expectation versus outcome.

(Paul, CM1)

### 3.4. Unworkable Strategies to Alleviate the Choke

Athletes’ reflections highlighted unworkable strategies they engaged in to alleviate the choke—either within competition as a means of recovery to ameliorate its impact on the remainder of their performance—or after, to avoid future experiences. This theme is comprised of five sub-themes (see Table 1).

Choking was conceptualised as a result of performers’ attempts to exert control over aspects of their experience and environment in order to facilitate optimal performance—including attempts to control their emotional experiences under pressure. This sub-theme included efforts of performers to overcome challenging experiences through “false positivity,” or “replacing negative thoughts with positive,” or grappling with “rationalising thoughts” in competition:

I had a lot of input from a few of the coaches, but I wouldn’t say they were very well-trained in handling that sort of thing…one of them said, ‘oh you just need to stop being negative, you need to stop thinking so negatively, of course you’re going to do bad if you say you don’t want to do it, you say that you can’t bowl.’ But, I was trying to tell them I was just being realistic, I knew what I could and couldn’t do, and actually, trying to be positive when you’re so low in confidence just doesn’t help, so I found almost that fake positivity just really didn’t help.

(Izzy, IF2)

This notion was also captured in the reflection of a golfer who highlighted the futility of fixating on the control of inner experiences through the use of an analogy:

In the summer, I ride motorbikes and there’s this thing they talk about, they call it target fixation. If you’re looking at an obstacle and say, ‘oh, I must avoid that obstacle,’ you end up driving *into* the obstacle. I don’t know if that’s what’s happening in my head, but I’m saying to myself, ‘for goodness’s sake, *don’t* over swing here, Paul! And of course, ((laughs)) you just end up doing exactly what you *don’t want to do*.

(Paul, CM1)

Also contained in this theme was the practice of increasing effort under pressure as a control strategy. The performers’ responses to pressure was reflected by a tendency to abandon routines and processes that had previously served them in competition, as under high-pressure conditions, they experienced an attachment to thoughts doubting that their previously relied-on practices would be sufficient. Inner experiences of over-thinking or dwelling on upcoming performances were responded to with examples of compensatory overefforting such as overtraining and ‘trying too hard.’ These behavioural responses may be reflective of the consequences of a ‘threat mindset’ and ‘low self-trust,’ discussed in theme two: conditions for the choke.

...Another thing [coaches] said is, ‘oh you just need to bowl loads and loads and loads and get back into it,’ and also, that didn’t work. At one point I was bowling like five times a week for...so many bowls, like up to 60 bowls five times a week and that’s like what, 300 bowls a week? And it still wasn’t getting better, so definitely, just repeating and repeating, the more I did badly as well the more of it I did, so actually just doing loads of it didn’t help.”

(Izzy, IF2)

It was common practice for participants to recall responding to discomfort with increased control, via increased effort or ‘doing more:’

I’ve tried to—as pressure increases leading up to the competition—whether that’s like through like your coaches being like, ‘all those meets coming up!’...kind of like overcompensate for the pressure, and it makes you panic and think that you’re not doing enough. So that’s why I feel like I changed my routine. I’ve got to do all of *this, this, this* and then that just kind of like adds to the stress, adds to the pressure. It’s like, ‘well, I’ve done *all of this* so *I should be doing well*,’ but I was doing just fine before in training. And then, when it- when I start changing everything, that kind of disrupts everything.

(Jamie, NF2)

This quotation illustrates how athletes often sacrificed ‘what works’ (functional processes such as pre-performance routines which had previously supported optimal performance) in the service of alleviating discomfort from thoughts such as ‘have I done enough?’ that commonly arose under pressure. Choking was also conceptualised as a consequence of restricting behaviours under pressure as a control strategy. Athletes reflected on limiting actions in these contexts to avoid a choke:

I have my spin ball [type of cricket bowling technique] and then I have one that just comes out the front of my hand. And I find that one that comes out the front of my hand I *don’t* choke with, I can put it on a spot and it can be fine. But it’s my spin ball that I’ve choked with. Sometimes if I lose my trust in that, I’ll just bowl the other bowl, so I can get out of the over, bowl it, but it’s like I can’t trust it—because the spin bowl is the one I’m actually *meant* to bowl the majority of the time, so…I just lose my confidence with it and go, ‘I’ll just bowl that to get out of the over.’

(Izzy, IF2)

Avoidance was identified as the dominant coping strategy under pressure. This included a tendency to prematurely end performance in the interest of escaping an anxiety-provoking experience. For example, several participants recalled rushing through routines or processes to get their performance ‘over with,’ often citing that they were preceded by feelings of low confidence, low trust, or high self-doubt in their ability to perform. Through a functional contextual lens, these findings demonstrate attempts by athletes to not only escape the competitive environment, but also, possibly their own internal experience of anxiety. This experience was juxtaposed with functional performance behaviours by one participant:

If I’m doing well, if I’m confident, I’ll act confidently…take my time a little bit more, organise my fields the way I want it, have a couple bowl-throughs to the person next to me, get my shoulders ready…take my time and feel confident. I think if I’m doing *badly*, like when I did badly, I rushed through it because I want to get this done as soon as possible. I want to get this done and go outfield. I’d rush through, I wouldn’t organise my field and I’d rush just to get it done. ‘Cause like, I had *that little confidence* that it was going to go right.

(Izzy, IF2)

Engaging in behaviour under pressure that was inconsistent with participants’ values as a performer was another consequence for athletes when attempting to alleviate choking. Participants reported a disconnection from values or their performance identity and performing in a way that was motivated by avoidance (of the choke or failure) rather than pursuing meaningful action (e.g., performance or process goals, embodying elements of their performance identity such as embracing risk). One international-level weightlifter reflected on avoidance—discounting the importance of competition and acting in a values-inconsistent way (e.g., identifying himself as someone who ‘wants to perform,’ while approaching competition with indifference under pressure):

I’d put the sort of feelings of emptiness and not wanting to like really lift and not feeling the *need* to have to make lifts as my choke. I’m someone who *wants* to do well and *wants* to perform to my highest standard every competition. So, to have these moments where *I don’t care* or *don’t want* to make this or, doesn’t matter if I make or miss it...I’d say that would be like a choke for me. And that was definitely what happened at the [redacted] Championships.

(Calin, IM1)

## 4. Discussion

This study is the first to explore the experience of performing under pressure through a functional contextual lens. The four main themes address how this performance phenomenon can be understood through a functional contextual approach. Importantly, this approach centralises the notion of changing the context surrounding behaviour as opposed to its form, through symbolic relating. This is believed to be facilitated through an enhanced awareness of the unique contexts operating for an athlete that give rise to the choking experience, as well as athletes’ verbal behaviour of relating to these experiences. The dominant narrative in choking research has focused on performers’ cognitive and emotional states as explanative models (e.g., attentional challenges, and cognitive and somatic anxiety). This study demonstrates that shifting focus to performers’ behaviours of relational responding to both their internal and external experiences offers a more pragmatic way to both conceptualise and begin to propose solutions for the choking experience.

This research extends knowledge by suggesting that athletes’ overt/observable, private/internal and verbal behaviour provides a conceptualisation of how choking under pressure occurs. The ability to symbolically relate is evident in participants’ accounts of choking as they articulated past experiences in which their performance environments were laden with cues—for example, attitudes and advice from trusted individuals in their performance environment ahead of significant performances, the positioning of a ball on the green, etc., which they uniquely related to. Verbal behaviour suggests that the function of athletes’ physical and private behaviours is determined by their relating, and is influenced by the context this occurs in. RFT proposes that the relations athletes have made cannot be unlearned or deleted from their networks (e.g., this competition matters *more* than the last, I’m going to choke *because* I feel the same as the last time I choked, I *should* hit this well *because* there’s nothing stopping me, *I can only* perform well *when* I feel confident), and relational frame theorists would suggest that the way athletes relate must be the source of change. In order to achieve this, the context these occur in must be altered.

The personal narratives theme contributes new knowledge regarding how verbal behaviour of relating and participants’ internal context poses challenges when performing under pressure. Participants identified processes of absorbing their experiences into a sense of self, making predictions for their future, and fixating on challenging inner experiences (Hayes et al., 2004). This finding echoes propositions made by the ACT model that fixation on self-stories limits an athlete’s ability to respond to moment-to-moment changes in their environment (Hayes et al., 2004). Data support ACT literature which suggests that domination of future-focused cognitions precludes athletes from engaging with their surroundings in an open and engaged way as they fixate on the content of their future-oriented thoughts. Athletes showed a tendency to engage with potential outcomes, fears for the future, or imagined scenarios in a way “as if they were actually happening” (Luoma et al., 2017, p. 445) at the expense of present moment awareness. Taken together, this theme demonstrated that contexts of rigid, narrow, or inflexible perspectives of the self could underpin the choking experience. Deductive analysis revealed that these narratives parallel the notion of self-concepts or the conceptualised self where individuals tend to devote cognitive energy into constructing and maintaining a coherent sense of self (Hayes & Pierson, 1999). The ‘self’ is conceptualised as “a set of verbal behaviours that are central to how humans develop a consistent perspective” to view the world (Luoma et al., 2017, p. 163). Athletes engaged in storytelling, self-evaluations/judgements, and making predictions for the future, all of which are practices of relating to internal (thinking, emoting, remembering) and external experiences which influenced their future behaviour.

Processes of inflexible ‘selfing’ were identified in which athletes engaged in practices of rigidly labelling themselves as performers, which underpinned inflexible behaviours under pressure—for example, fixating on an identity as a choker, or a perfectionist, where responses often included disengaging from present-moment focus to change or control their thinking as a result of prescription to these concepts. The literature asserts that while not inherently problematic, fusion with a conceptualised self can become unworkable when individuals engage in behaviours to achieve coherence with this sense of self, at the expense of workable or adaptive behaviours (Hayes, 2002). Through a functional contextual perspective, where functional analysis is used to make sense of behaviour in context, it is logical to hypothesise that athlete behaviour was (at least partly) driven by responses to self-concepts to sustain a consistent sense of self (in the example of the footballer, withdrawing from the match as his self-concept dictated that he was intolerant of suboptimal performances). In this sense, athletes who place emphasis on their perceived self-concepts may be inadvertently restricting their behaviour, engaging in actions that cohere with a self they rigidly identify with. This contribution of knowledge suggests changing the context of athletes’ relating (‘this is just a story I tell myself,’ or ‘I am the container/holder of these perfectionistic thoughts, but I *myself* am not the thoughts’) holds promise for changing behaviour, as opposed to investing mental resources into eliminating anxiety experienced in the form of self-labels, stories or self-concepts as a prerequisite for optimal performance.

Extending this notion, the understanding of the choking experience was further progressed through deductive analysis which captured practices of athletes over-identifying with their internal experiences, absorbing these into the sense of self. In ACT terms, this is articulated as ‘self-as-content’ and is reflected by athletes deriving a sense of self from the amalgamation of these experiences (I am what I *think*, I am what I *feel*, I am what I *do*), as opposed to acknowledging these experiences as distinct from the self and part of the ebb and flow one expects to experience as a sportsperson (for example, ‘*I am having the thought that* I am useless,’ vs. ‘I am useless’). This encapsulates the tendency for people to become entangled in the content of their internal experiences (thoughts, emotions, memories, evaluations and judgements)—known in ACT as cognitive fusion—to the extent that they become unable to respond flexibility. Cognitive fusion challenges athletes as they identify *as* their experiences, or view their world *through* them. This is in opposition to adopting an observer-like stance towards their experiences, with the ability to view the world *from* a *defused* perspective, by looking *at* their thoughts. This perspective increases the likelihood of perceiving thoughts for what they are, just thoughts—or from a further defused perspective, a collection of letters and words—and when verbalised, just a collection of sounds (Luoma et al., 2017). This conclusion offers support for assessing the ‘workability’ of behaviours when performing under pressure. Importantly, this conceptualisation offers a more pragmatic approach as it frees athletes and practitioners up from unrealistic demands of reducing or eliminating private events (e.g., negative thinking, anxiety in anticipation of an upcoming performance), and instead, shifts the goals of work to assessing the workability of them through functionally analysing the resulting behaviours. For example, practitioners can help support athletes in developing a contextual perspective of themselves when performing under pressure (for example, ‘instead of allowing a single shot to define you as a golfer, what if you instead considered the identity you wish to evoke and the presence you’d like to exude when you face a critical shot’). These experiences are further conceptualised by the notion of ‘self’ within RFT, particularly the concept of self-discrimination as the ability to differentiate one’s responding from their sense of self. Additionally, the ability to engage with ‘healthy’ or adaptive selfing is also reflected in four selfing processes and achieved through a combination of self-as-process and self-as-context engagement (I as *various*, I as *flexible*, I as *container*, I as *perspective*) (McHugh et al., 2019).

ACT exercises, which have been found to be efficacious in the support of individuals with depression and anxiety, suggest that through supporting athletes to establish a ‘transcendent sense of self,’ they can develop an awareness that they are more than the actions they perform when competing, not limited or defined by the feelings or thoughts they experience under pressure, and are instead a stable observer of their own experiences. This conceptualisation of behaviours underpinning the choke suggests that changing the context athletes experience these private events in (for example, training athletes to approach their thinking with a posture of non-judgmental awareness, and, through hierarchical relational framing, be able to perceive themselves as observers of their thinking) has greater impact on behaviour than attempting to change internal experiences. This is particularly true for athletes who interpret internal and external experiences as indicative of an impending choke. Instances where athletes referenced physiological sensations during performance (“tightness in my throat,” “feeling tired when I know I’m rested”), which they interpreted as having a direct impact on subsequent behaviour (“it’s [a choke] going to happen again”), is potentially indicative of challenges in selfing processes. In these examples—through an RFT lens—participants ignore the context these psychophysiological responses are situated in, paying attention to only the relation: ‘I felt this way last time I choked, so I know it’s coming’ (equivalence, causal and temporal relational frames), based on their symbolic learning history. In these scenarios, insensitivity to context precludes the athlete from interpreting these sensations in different (more helpful and adaptive) contexts; for example, in a context of non-judgmental awareness, such sensations may signal a readiness to perform, or as a trigger to initiate pre-performance routines to help regulate arousal and defuse from cognitions prior to competition.

Athletes’ reflections on the conditions that gave rise to their choking experiences provide evidence for a property of relational framing that practitioners can manipulate to influence behaviour change through a process known as transformation of stimulus functions. Athletes reported various conditions in which they experienced a choke, which are believed to be underpinned by and explained through relational frames. For example, a golfer captured a reoccurring theme when reflecting on choking in contexts of ‘this matters,’ which an RFT theorist may hypothesise a frame of distinction is operating to influence behaviour. Similarly, a cricketer interpreted her own anxiety ahead of competition as a cue signalling an impending choke through comparison to sensations experienced during past choking events. In these examples, RFT would suggest arbitrarily applicable derived relational responding (AADRR), or more specifically, frames of comparison or causality may be used to understand the subsequent behaviours and choke. These instances illustrate how language enables individuals to transmit their own past experiences “to make useful predictions about the future” (Bennett & Oliver, 2019, p. 32). The challenge presented in the choking accounts, however, is that predictions aren’t always ‘useful’—as athletes use past experiences to interpret the consequences of experiencing certain emotions or thoughts during or prior to competition (e.g., feeling or thinking ‘x’ will lead to performance failure), instead of allowing their internal experiences to ebb and flow without an attachment to them. RFT theorists might suggest that to influence choking behaviour, a practitioner must change the stimulus function; that is, instead of athletes responding to coach instruction, the perceived importance of an event, or internal rules by abandoning process or changing behaviour in other maladaptive ways, these stimuli can serve a new function of prompting athletes to commit to their well-rehearsed routines and an implicit reminder to trust their abilities.

This theme encapsulated various forms of relating believed to underpin the choking experience, for example, temporal or causal framing, as athletes indicated that the presence of certain thoughts or emotions was indicative of a choke (‘*if* I lose my confidence, *then* a choke is bound to happen’). Additionally, participants who shared insight into similar experiences of the symbolic importance of a game (i.e., verbal behaviour indicating that it’s ‘known’ that the game is ‘too big for you’) may demonstrate comparative relational framing and cognitive fusion. These processes of inflexibility may have precluded athletes from engaging in flexible thinking, and instead, given them a fixed perspective in which they become entangled in disputing the ‘truth’ of these beliefs. Importantly, participant accounts suggested that frames of comparison and frames of distinction were among the most prominent and influential forms of responding. Symbolic relations performers drew between their past selves (for example, previous performances, a performance feeling ‘too big’ for them) and relations regarding the distinctiveness of events (e.g., the symbolic enormity of the event in terms of importance) suggest that these relational frames are worthwhile functional relational units to explore in future research.

Through an RFT deductive lens, the study findings provide evidence for rule-following or ‘rule-governed behaviour’ as a form of verbal behaviour. While rule-following has been shown to accelerate learning and prevent humans from contacting negative consequences (e.g., contacting and following the rule in rugby: ‘a player cannot be tackled in the air’ reduces the likelihood of injury for either player), when behaviour becomes excessively controlled by rules, athletes become insensitive to direct contingencies and changes in the environment (Hughes & Barnes-Holmes, 2015). When thoughts, rules, reasoning, or instruction alone govern behaviour without direction from environmental feedback, athletes preclude themselves from opportunities to make decisions based on ‘what works,’ and fall into traps of rigid and inflexible behaviour (Bennett & Oliver, 2019). Importantly, evidence from this theme suggests that athletes who have choked may be engaging in rigid rule-following behaviour, which prevents them from adapting to changes in their performance environment. Findings revealed athletes often held implicit rules for themselves, for example, having to control their anxiety in order to achieve optimal performance or self-imposed expectations for success functioning as self-rules. These examples suggest the presence of rules operating for athletes who have choked, specifying conditions for performance such as, ‘I can’t perform well when I’m feeling this much anxiety,’ or ‘there’s nothing stopping me, I should do well here.’ From an RFT perspective, the key consequence of this verbal behaviour is that, when it is applied to contexts where it is ‘unworkable,’ maladaptive behaviours often result. For example, RFT can be used to explain that through persisting in the anxiety-provoking situation, the athlete can contact a useful consequence: learning through experience that performance is not contingent on the presence/absence of emotions, but rather, how they are responded to, and what one can achieve by resisting avoidance or “safety-seeking behaviours” (Bennett & Oliver, 2019, p. 198). In the context of performing under pressure, resignation in the presence of anxiety is not workable; in the short-term, the athlete may experience immediate relief as they avoid prolonged experience of the uncomfortable thoughts, emotions and sensations associated with performance anxiety; however, following this rule precludes them from contacting long-term consequences (e.g., persisting under pressure, testing their skill in a high-stakes match while under fatigue, and ultimately, performing in a values-consistent way). In this example, and across many participant accounts, choking was conceptualised as a consequence of unworkable rule-following. Through a causal frame, an athlete relates to their anxiety: ‘when I feel anxious, I can’t perform,’ and in response, engages in experiential avoidance (e.g., rushing a routine, asking for a substitution) in efforts to eliminate their anxiety to facilitate performance. Importantly, practitioners who identify rule-governed behaviour can address this tendency through ACT processes including defusion strategies (for example, taking the literality out of language: asking an athlete to make an ‘I can’t statement’ and then asking them to perform the action they stated they couldn’t) or assessing the workability of expectations for perfection (“what do you gain from playing ‘perfectly’...how are you able to live by your value of ‘finding ways for continual improvement’ if every performance is flawless?”).

The ‘unworkable strategies to alleviate the choke’ theme offers insight into the unworkable behaviours that practitioners can replace with practices in psychological flexibility to impact the choking experience. A large proportion of athletes’ responses to performing under pressure provided evidence for the ACT inflexibility process, ‘experiential avoidance’ (for example, abandoning processes, restricting involvement to avoid errors, or exert control through overplaying/preparing). Through a relational frame lens, participants’ reflections on their practices of limiting actions under pressure—responding to feelings of anxiety and panic with avoidance or resignation—can be conceptualised as a narrowing of behavioural response repertoires, that is, behavioural responses available to an athlete based on their learning history. From an ACT perspective, this behavioural narrowing is problematic as avoidance behaviours often divert athletes’ focus from pursuing meaningful or value-driven behaviour, for example, expressing themselves or persisting under pressure (Bennett & Oliver, 2019). From an RFT and ACT perspective, psychopathology is conceptualised as “a narrowing behavioural repertoire that develops over time through maladaptive strategies to cope with unwanted experiences” (Webster, 2011, p. 309). In this same way, choking could be conceptualised as an experience where the overextension of language (i.e., language in the context of literality, attempting to get rid of suffering through problem-solving) functions to reduce the “healthy functional variation” in behaviour and one’s “ability to respond adaptively to internal and external environments” (Luoma et al., 2017, p. 16). In the sporting environment, where athletes experience fear, self-doubt, anxiety, and panic in contexts of fusion with thoughts and avoidance of emotions, inflexible patterns of behaviour result. From a CBS perspective, this occurs due to “intolerance of internal distress” (for example, a lack of acceptance or willingness to experience challenging inner experiences) and a “strong reliance on verbal rules” (as discussed in the ‘rules for choking’ theme) (Bluett et al., 2014, p. 613). Under pressure, athletes reported responding rigidly to their internal experiences—many of which have been discussed in the personal narrative theme—for example, inflexible attention, cognitive fusion, disconnection from values, attachment to a conceptualised self, and avoidance. Importantly, athletes’ accounts also suggest there could be value in utilising acceptance-/mindfulness-based approaches for facilitating performance under pressure. For example, a cricketer reflected on the internal battle of allowing herself to sit with the negative imagery of her bowling poorly, or giving into an urge to avoid it by replacing it with positive visuals (e.g., an example of an avoidance strategy aligned with the eliminative agenda of traditional second-wave cognitive-behavioural practices). This was also reflected in the weightlifter’s account of adopting a present focus to execute her pre-lift routine. Taken together, evidence from athletes’ accounts suggests there is merit in exploring how changing the context of challenging experiences—which are inevitable during performance under pressure—offers a potentially viable new approach for resolving performance failures.

### 4.1. Strengths and Limitations

This study intentionally omitted a definition of choking under pressure in the recruitment of its participants. This research decision is a strength as the participants’ experiences were kept at the forefront of the investigation. This ensured the mechanistic narrative of choking was not perpetuated as this would stifle novel findings, including insight into participants’ individualised contexts surrounding the event, which was of paramount interest. The methodological decision to leave the operationalisation relatively open-ended at recruitment allowed the participants’ experiences to be explored through an interview schedule that permitted the broader context surrounding the choke to be captured. Revisiting the phenomenon in this way also offers a considerable strength by ‘getting close’ to the research from those who have rich experience, and was highlighted as a key requirement for efforts undertaken that seek to take on the seismic challenge of revisiting the choking definition and conceptualisation (Mesagno & Hill, 2013).

Another strength was the methodological decision to capture focus-group behaviours. In line with CBS research, all behaviours (verbal/covert or observable/overt) are valuable for understanding the athlete experience of choking under pressure, therefore capturing participant physical behaviour that often deepened insights from verbal behaviour (for example, nervously laughing, covering one’s face with their hands or speaking quickly to avoid drawing out a reflection of performing poorly) allowed the study to be rigorous and consistent with CBS methods. For example, it was common for participants to communicate their experience through physical gestures alluding to an obstructed perspective of their world during their choking experience, for example, hands in front of the face illustrating the impact of thoughts as ‘the red mist.’ This gesture and similar ones parallel a popular metaphor in ACT (‘hands as thoughts and feelings,’ Harris, 2019), where the practitioner asks the client to obstruct their vision with their hands, and then invites them to gradually distance them by extending their arms, while asking the client to reflect on the ease/difficulty they experience with respect to engaging with the world around them. To this end, participants articulated this same notion of cognitions impeding one’s ability to meaningfully engage with their environment. This metaphor also alludes to the function of these behaviours under pressure, narrowing their perceptual field, and as a consequence, adaptive behavioural responses through disengaging from the moment-to-moment experience of the match by opting instead to engage in an internal battle with their own self-judgements.

A final strength of the study lies in its methodology which adequately addressed guidelines made by the CBS task force—a group of CBS researchers who have stipulated criteria and recommendations to ensure the production of quality CBS research (Hayes et al., 2021). In line with the assertion that functional contextual research is process-based, the task force has called for more basic behavioural research to identify processes of change and build practical applications (in this context, choking interventions; Hayes et al., 2021). Importantly, this study represents a necessary first step in deriving meaningful insights through qualitative choking research from a functional contextualist perspective to lay the foundation for future experimental and intervention research. Overall, these strengths highlight the value of this piece of exploratory research to broaden the scope of how the choking experience can be conceptualised, and provide the foundation from which to develop pragmatic strategies to facilitate optimal performance experiences in future applied research.

It is acknowledged that the sample was small and heterogeneous, at the levels of skill, sport and gender; however, the priority was to achieve homogeneity at the level of the choking experience. The heterogeneity is a limitation to the study, as varying sports and performance levels could dilute the depth of data and create challenges with respect to “meaningful core cross-case themes” (Robinson, 2014, p. 4). As not only athletes, but human beings who behave linguistically (Ciarrochi et al., 2005), the researchers have reconciled this as a trade-off in the interest of achieving greater understanding of the shared behavioural elements of choking derived from athletes performing across varying sports, levels and contexts, to potentially shed light on how processes of symbolic relating transcend the differing expressions of choking in sport. Retrospective recall of choking experiences also posed a limitation to the study. Several participants recounted experiences that occurred several years in the past which could have resulted in recall bias. Participants relied on memory, which may have compromised the accuracy of their reflection to a greater extent than if they had occurred in the more recent past. To adequately address this concern, future research could look to supplement interview data through methodological triangulation, for example, the inclusion of observational or ‘think aloud’ data to enhance and corroborate participant recall. While these factors could improve future research, it is also acknowledged that focus groups provided a context where “synergistic sparking off” occurred as participants engaged with responses offered by their peers to strengthen recall and mitigate challenges of memory and reconstruction (Cleary et al., 2014, p. 474). An additional element of the focus group interviewing that potentially limited the strength of the findings was the virtual nature of the majority of the interviews. In line with RFT and CBS theory, which recognises the importance of capturing observable behaviour as crucial for understanding how it can be predicted and influenced during performance experiences, facial expressions, gestures and other non-verbal behaviours were captured for analysis. The virtual medium of data collection was a limiting factor in capturing and identifying this behaviour, as it could be argued that more subtle responding (e.g., countenance, utterances) was potentially undetected. Future research would be well-informed to conduct all interviews in person to overcome this limitation.

### 4.2. Implications for the Conceptualisation of Choking

The literature to date has narrowly defined choking as an ‘outcome’ of attentional mechanisms (self-focus, distraction), ignoring the more contextual moment-to-moment behavioural changes that have something important to say about how athletes symbolically relate to their internal and external experiences. The present findings suggest choking is a complex phenomenon that must be conceptualised beyond outcome and further differentiated as a unique and contextually driven performance ‘*experience*.’ It is argued that ‘performance experience’ practically encapsulates the context and behavioural response sequence of individuals’ performance under pressure, recognising that pressure itself has a unique learning history. From this perspective and given initial insights emerging from this exploratory study, practitioners are tasked with explicating symbolic relations and contextually driven behavioural responses at the individual level to understand how to facilitate optimal performance, rather than relying on broad causal mechanisms to develop interventions to combat. This conceptualisation is believed to lend itself more pragmatically to intervention designs that function to change the contexts around behaviours which often precede and reinforce choking-related behaviours, instead of eliminating the behaviours themselves. Several participants made reference to performances where the ‘process’ of performance was compromised yet the ‘outcome’ was maintained or favourable—either through random chance, or based on context the behaviour was situated in (for example, in the cases of the cricketer, golfer and weightlifter who made references to their performances appearing favourable or onlookers not being privy to the performance breakdown despite behaviours indicating a choke had occurred). These findings highlight the importance of context in conceptualising choking as it cannot be solely determined by observable outcomes of success or failure, but also through relating to one’s internal experiences and the consequences for overt behaviour.

The notion of a breakdown in process when performing under pressure also relates to the novel concept proposed in this study of the ‘choking experience,’ and was supported by athlete reflections. This finding contributes to the extant literature by challenging previous conceptions of choking that argue it is mechanisms—for example, anxiety—that are the causal drivers of attentional errors including self-focus or reinvestment which condition a choking outcome. The study findings reported here extend contextual behavioural thinking by providing experiential accounts suggesting that it is not the mere presence of challenging inner states, but rather the way athletes respond to these states that dictates whether a choke ensues. This perspective therefore posits that it is possible to achieve optimal performance in the presence of challenging inner experiences, by transforming their function on behaviour (White et al., 2021). Athletes who reported fixation on the possible consequences of choking, self-criticisms or judgements in response to failed rule-following before performance, and as a result, adjusted behaviour, demonstrate how a choking ‘experience’ is worth differentiating from a choking ‘outcome’ (an acute and observable performance breakdown that has been traditionally associated with choking). In the former, performers may be challenged with difficult inner experiences that they respond to through behavioural changes (e.g., avoidance through increasing the speed of their pre-shot routine). Participant accounts strengthen the functional contextualist argument that it is the way these are responded to (through the human ability to relationally frame) that dictates if these experiences translate to a choking outcome, as opposed to their mere presence indicating that a choke has occurred. This notion was supported by participants who reflected that often “all the same emotions are there” (during high pressure competition when a choke, or an optimal performance results). Through a CBS lens, the human ability to symbolically relate and relationally frame offers a potential missing link between athletes’ internal states, and whether a ‘choking experience’ (behavioural changes in response to internal/external experiences) or ‘outcome’ (traditional observations of performance success/failure) ensues. These conclusions have implications for applied practice, as practitioners may help athletes equip themselves with skills in psychological flexibility that will facilitate changing the context of, and their response to, challenges within the performance experience.

The literature on choking has been constrained by a lack of consensus over definition, and these inconsistencies have also been evident in real-world settings. This has presented challenges for research and practice alike, and it is proposed that a practical way forward is to retain functional and behavioural aspects of existing choking definitions, such as those originally put forward by Baumeister (1984) and Baumeister and Showers (1986), where there is a greater focus on observable behaviours. The findings combine to challenge existing notions of choking and assert that by applying a functional contextual lens, it can be more usefully conceptualised as a ‘performance experience’ that comprises unhelpful narratives and ways of relating to one’s private experiences and rigid rule-following, and is a product of attempts to control one’s experience through unworkable strategies. Understanding choking through patterns of symbolic relating proposes a novel approach for future behaviour-change interventions through identifying the unique symbolic relations athletes have absorbed into their relational networks and constrained their behavioural response repertories for performing under pressure, and subsequently, training athletes in the processes of psychological flexibility. While future experimental research is required to build on this exploratory investigation, it is proposed that identifying the unique relational frames that make up an athlete’s experience of choking is recognised as a potentially crucial first step to understand how they currently function to restrict behaviour. Once this is identified, practitioners are believed to be better positioned to transform the function of choking-related inner experiences to promote more adaptive performance experiences and outcomes.

The challenge in recruiting participants through athlete gatekeepers is believed to be reflective of the pervasive processes of relating within elite sport which have implications for future study of the choking phenomenon in sport. In several instances, gatekeepers (e.g., coaches, performance directors, team managers) were apprehensive about allowing their athletes to participate in focus groups, as it was insinuated that discussing this experience would have costly implications for athletes’ mindset ahead of important upcoming performances. It is suggested that these anecdotes may unintentionally provide topical evidence of the pervasiveness of relational framing in everyday language and how it can function to restrict behaviour when it is overextended. Gatekeeper’s apprehension surrounding their athletes’ participation in the research—where they are assumed to be recounting experiences of, and reflecting on, choking under pressure—is perhaps reflective of causal and temporal frames in operation (*if* they talk about or actively recall the choke *now*, *then* perhaps that will *cause* their performance to be negatively impacted *at the weekend*) or frames of coordination (talking about choking *is like* tempting fate, or, *is like* setting yourself up to choke again). This underscores a necessity to centralise the concept of relational framing and responding in the applied world, to dispel unhelpful practices off the back of symbolic relating and relational framing. This highlights a need to expose the consequences associated with the unhelpful proliferation of symbolic relating. Additionally, it recognises the importance of changing the context around these experiences, for example, facilitating awareness of contextual cues present in an athlete’s performance environment, including training in how they could be better equipped to respond to them rather than falling victim to them.

### 4.3. Practical Implications

The present findings provide preliminary insight into a functional contextual approach that sport psychology practitioners may thoughtfully adopt to functionally analyse an athlete’s experience of choking. While the present study is restricted by its exploratory design to make causal statements with respect to third-wave approaches for athletes who have choked, the findings suggest there is merit in utilising contextually sensitive and pragmatic methods for addressing concerns of choking under pressure by first identifying the contextual cues (e.g., situational, environmental triggers such as language, visual cues) that impact on performance. Insight from the study’s RFT framework suggests that identifying these factors may allow practitioners to conduct functional analyses that parse apart the relational (what an athlete *thinks* based on the relations derived) and functional (the *psychological impact* of what they think) contexts, which may inform how they could approach changing the context around these experiences. These perceived benefits of approaching choking from a functional contextual perspective include equipping practitioners with the knowledge of which relations they can evoke internally (as opposed to recreating the exact conditions of a pressurised environment) in order to allow athletes to practice responding to these cues in a more adaptive way. For example, a practitioner may target changing a golfer’s maladaptive behavioural response to a leaderboard from avoidance, to striving, through transformation of stimulus functions. The practitioner may initiate this work by first identifying the environmental cue which—based on the athlete’s unique symbolic learning history—provokes internal experiences of being compared (relational frame of comparison) and consequently, evokes functions of avoidance (rushing his pre-shot routine to get the shot over with and escape prolonged contact with the leaderboard).

The practitioner is then tasked with recreating internal environments that elicit ‘comparison’ (for example, within training drills, evoking comparisons to their past self) in which they may utilise processes of present moment awareness, acceptance of uncomfortable emotions, and also engagement with values for performance that are meaningful for the athlete in order to change the *context* around the *experience* of comparison. As a result of changing this behavioural sequence, when the athlete contacts relational cues of comparison in his performance environment (e.g., leaderboard, public rankings or seedings, thoughts of ‘I’m being compared’), behaviour is selected as a consequence of the stimulus function being transformed into striving for optimal performance (e.g., *I want to stay in the moment to do my training justice, connecting with an identity of being composed under pressure*), rather than avoidance, (e.g., *I can’t look at this, I need to escape*) to facilitate behaviour change under pressure. The exploratory study findings therefore propose that there is potential utility in identifying the various relational frames that are operating for athletes at any one time during competition under pressure (e.g., causal if/then statements or beliefs), as well as any behaviours of rule-following (‘I should’). This represents a crucial first step for understanding how these features fit within an athlete’s broader relational network, including how this has consequences for performance and how practitioners could approach understanding these networks to help build adaptive and more contextually sensitive patterns of behavioural response repertoires.

### 4.4. Future Directions

Based on the present findings, there are several meaningful areas that could be pursued through experimental research. Based on the identification of various relational frames proposed to be operating for athletes, developing an empirical study that tests the effects of frames on behaviour during pressurised performance would be a useful research endeavour to draw conclusions on the impact of frames that reliably provoke choking behaviours. Additionally, researchers could build on this study by exploring the impact of rule-following on performance. Several styles of rule-following were identified (for example, tracking, pliance) that impacted performance. It may be necessary to first explore this through qualitative methods to unearth richer themes of rules and rule-governed behaviours prior to experimental research on their impact on performance behaviours.

The Association of Contextual Behavioural Science (ACBS) task force has called for more applied behavioural research to identify processes of change in psychological research. It is suggested that building from the contribution of this study regarding relational framing and responding, future experimental research should make use of intensive longitudinal designs to gain rich data on the highly individual processes of experience, symbolic learning and behaviour change, for example, via single-case experimental designs (SCEDs) using techniques including ecological momentary assessment (EMA) or experience sampling methods (ESM) (Hayes et al., 2021). Logically following recommendations for experimental research, the present findings lay the foundation for intervention studies that can make use of bespoke third-wave (e.g., RFT- and ACT-informed) interventions to target problematic patterns of behaviour, through processes of transformation of stimulus functions, rule-following and relational framing that have been introduced in this study.

## Figures and Tables

**Table 1:** Summary of key characteristics and sub-themes for master themes.

Master Theme	Sub-Themes	Characteristics
Personal Narratives of Choking	Attachment to self-stories; Fixation on inner experiences; Preoccupation with the future; Athletes constructing a fearful environment; Consequence of inflexible attention; Choking is a performance experience; Psychologically flexible responses to pressure are possible; Belief that a choke is the result of failure to adaptively respond to ‘warning signs’; Belief that choking is out of one’s control; Choking is about how you relate to what you experience	The stories and descriptions athletes attach to the performance experience of choking under pressure
Conditions for the Choke	Context of ‘this matters’ created around the event; Consequences of low self-trust; Threat mindset around competition; Relating to anxiety as ‘bad’ predicts maladaptive behaviours; Historical contexts facilitate the choke; Salient contexts and cues for the choke; Belief that coordinating past and present experiences can protect against the choke; Conditions on one’s ability to perform; Perceptions of hopelessness	The conditions that give rise to the choke, including the way athletes symbolically relate to their experiences of performing under pressure
Rules for Choking	Consequences of rule-following; Negative effects of language in the performance environment; Expectations	Behaviours of rule-following that govern the choke, including implicit and explicit rules or expectations from athletes themselves or external sources
Unworkable Strategies to Alleviate the Choke	Fixation on control in the performance environment; Behavioural response repertoire narrowed under pressure; Avoidance as the predominant coping strategy; Unworkable behaviours as a long-term consequence of choking; Values-inconsistent behaviour under pressure	Athletes’ experiences of attempting to alleviate the experience of, or overcome the choke

## Data Availability

Data are available upon reasonable request from the authors.

## Abbreviations

The following abbreviations are used in this manuscript:

CBS	Contextual Behavioural Science
RFT	Relational Frame Theory
FC	Functional Contextualism
AADRR	Arbitrary Applicable Derived Relational Responding
ACT	Acceptance and Commitment Therapy
ACBS	Association for Contextual Behavioural Science
EMA	Ecological Momentary Assessment
ESM	Experience Sampling Method
SCED	Single-Case Experimental Designs
